# SARS-CoV-2 Infection and the Liver

**DOI:** 10.3390/pathogens9060430

**Published:** 2020-05-30

**Authors:** Katie Morgan, Kay Samuel, Martin Vandeputte, Peter C. Hayes, John N. Plevris

**Affiliations:** 1 The University of Edinburgh Hepatology Laboratory, Division of Heath Sciences, University of Edinburgh Medical School, Chancellor’s Building, Edinburgh BioQuarter, 49 Little France Crescent, Edinburgh EH16 4SB, UK; M.F.R.Vandeputte@sms.ed.ac.uk (M.V.); P.Hayes@ed.ac.uk (P.C.H.); J.Plevris@ed.ac.uk (J.N.P.); 2 The Jack Copland Centre, Advanced Therapeutics, Scottish National Blood Transfusion Service, 52 Research Avenue North, Edinburgh EH14 4BE, UK; K.Samuel@ed.ac.uk

**Keywords:** SARS-CoV-2, COVID-19, liver, drug induced liver injury, microthromboses, viral damage

## Abstract

A novel strain of coronoviridae (SARS-CoV-2) was reported in Wuhan China in December 2019. Initially, infection presented with a broad spectrum of symptoms which typically included muscle aches, fever, dry cough, and shortness of breath. SARS-CoV-2 enters cells via ACE2 receptors which are abundant throughout the respiratory tract. However, there is evidence that these receptors are abundant throughout the body, and just as abundant in cholangiocytes as alveolar cells, posing the question of possible direct liver injury. While liver enzymes and function tests do seem to be deranged in some patients, it is questionable if the injury is due to direct viral damage, drug-induced liver injury, hypoxia, or microthromboses. Likely, the injury is multifactoral, and management of infected patients with pre-existing liver disease should be taken into consideration. Ultimately, a vaccine is needed to aid in reducing cases of SARS-CoV-2 and providing immunity to the general population. However, while considering the types of vaccines available, safety concerns, particularly of RNA- or DNA-based vaccines, need to be addressed.

## 1. Introduction

A novel strain of coronaviridae (SARS-CoV-2) was first reported in the Wuhan province of China in December 2019. As of 8 May 2020, it has spread to 215 countries with 265,961 deaths worldwide [1]. On 11 March 2020, the World Health Organisation categorised the outbreak as a pandemic [2,3].

The SARS-CoV-2 virus is a single stranded RNA, enveloped, beta coronavirus characterised by spikes protruding from the surface [4]. Normally found in mammals, birds, and reptiles, this strain has not previously been identified in humans [5]. Previous strains of coronavirus outbreaks in humans include Middle East Respiratory Syndrome (MERS) in 2012 and Severe Acute Respiratory Syndrome (SARS) in 2003 [5,6].

Similar to SARS, SARS-CoV-2 is primarily transmitted by respiratory droplets produced by infected persons when they sneeze, cough, or are deposited on surfaces, where they are transmitted through contact. However, as SARS-CoV-2 has been detected in the gastrointestinal tract, urine and saliva, other routes of transmission have been considered [7,8].

COVID-19 disease refers to infection with the SARS-CoV-2 virus. Incubation time is within 14 days following exposure, with a median of four days [7,9]. Although often asymptomatic (with frequency estimated between 17% and 88% of cases) [10,11,12,13,14], infection initially presents with a broad spectrum of symptoms that typically includes general malaise, fever (commonly over 37 °C), dry cough, shortness of breath, anosmia/dysgueusia, headaches, and muscle aches [7,11,15,16,17]. Some other viral related symptoms, albeit less common, can also be seen—sore throat, chest pain, nausea, vomiting, diarrhoea, skin rashes, and vasculitic manifestations. Severe infection seems to present a biphasic pattern [18,19,20,21]. A first phase (‘viremia’), corresponding to viral invasion of the body, causes symptoms as described above. This phase is followed by an ‘inflammatory’ phase, corresponding to excessive host inflammatory response (‘cytokine storm’), responsible for severe cardiopulmonary manifestations, sometimes leading to acute respiratory distress syndrome, shock, and death [18,19,20,21]. Respiratory symptoms, in particular hypoxia, have been the main indication for hospitalisation.

It has been reported that 14.8–53% of SARS-CoV-2 patients had liver injury indicated by abnormal liver function tests—mainly elevated alanine aminotransferase (ALT), hypoalbuminemia, and elevated gamma-glutamyl transferase (GGT) [22,23,24]. These abnormalities seem to occur during either the viremia or inflammatory phase. Reduced albumin can be due to inflammatory response while raised levels of GGT and bilirubin are associated with biliary damage. This is confirmed in recent reports that SARS-CoV-2 has a much greater affinity for biliary cells (cholangiocytes), which have higher expression of ACE2 receptors compared with hepatocytes [7,22,25]. Significant liver injury with raised levels of ALT, Bilirubin, variable levels of alkaline phosphatase and GGT has been reported in 58–78% of patients with severe clinical manifestations of COVID-19 disease, being a surrogate marker for adverse outcome [4,7,15,22,25] (Table 1).

This review summarises the up to date knowledge on liver injury in the context COVID-19 disease in patients with or without pre-existing liver disease. We also discuss possible mechanisms of liver injury and the current advice regarding management of liver disease patients including liver transplant recipients.

## 2. Viral Entry and Effect on Liver

SARS-CoV-2 enters the host via the Angiotensin-converting enzyme 2 (ACE2) receptor. It has been suggested that SARS-CoV-2 binds ACE2 receptors more efficiently than previous COVID viruses, allowing for its extensive transmission [26].

ACE2 is found in a variety of tissues (heart, liver, lung, bladder, kidney, and pancreas); however, it is known to be abundant in alveolar cells accounting for the viral injury to lungs of infected patients [22]. While there is conflicting evidence of ACE2 receptor density in the liver, current reports using single-cell RNA sequencing have confirmed that cholangiocytes have the highest levels of ACE2 receptors [7,22,25,27]. Hu et al. used in silico and in vitro techniques to sample hepatocytes, cholangiocytes, Kupffer cells and other components of fresh liver samples [25]. They found that 59.7% of cholangiocytes had ACE2 receptors in comparison to only 2.6% of hepatocytes. This data suggests that cholangiocytes have the same percentage of ACE2 receptors as aveolar type 2 cells [25]. Further, it has been suggested that infection of cholangiocytes may be the source of the virus found in faeces [28]. While the presence of a receptor is needed for the virus to gain entry into the host, it is still unclear if other conditions are also needed or could possibly aid the virus.

## 3. Possible Causes of Elevated Liver Enzymes

Emerging data for abnormal liver enzymes seen in SARS-CoV-2-infected patients raises several questions. Are these abnormalities due to direct viral damage, drug-induced liver injury (DILI), unknown pre-existing liver disease, or indirect consequence of viral damage to other systems (cardiopulmonary, haemostasis)? Liver samples from infected patients were examined, and moderate microvascular steatosis with mild lobular and portal activity were reported [29]. It does seem likely that damage that may affect liver function could principally be due to hypoxia and shock, although a direct effect of SARS-CoV-2 to the liver or DILI can also be contributing factors [29,30].

### 3.1. Direct Viral Damage

While mechanisms of direct damage to the liver remain unclear, concerns about viral damage have already been raised, e.g., with a case of SARS-CoV-2 infection concurrent with liver failure, without other apparent cause, recently described in Germany [31].

**Figure 1:**
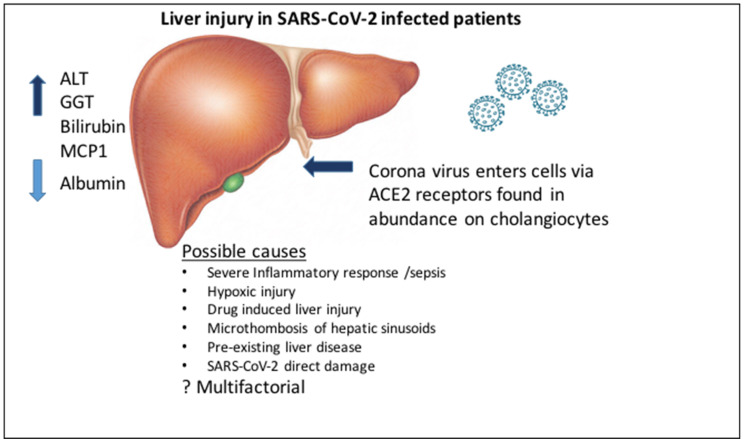
Liver injury in SARS-CoV-2. There are multiple reports of increased liver enzymes and liver dysfunction in SARS-CoV-2 patients presenting with elevated alanine transaminase (ALT), gamma-glutamyl transferase (GGT), bilirubin, and monocyte chemoattractant protein 1 (MCP1). Taken together with lower levels of albumin, this points to liver damage with possible injury to biliary cells. Liver injury is most likely multifactorial and seen mainly in patients at the severe end of the disease spectrum.

However, direct viral damage has been contested by some, and other explanations have been offered, which will be discussed below [30] (Figure 1).

### 3.2. Drug-Induced Liver Injury

A study by Fan et al. [29] has raised the question of DILI as a possible cause of liver injury seen in COVID-19 patients. They show that patients given lopinavir or ritonavir after admission presented higher incidence of liver injury and required longer stay in hospital. It is also possible that these patients were given antivirals because they had a more severe presentation that might have affected their liver in the first place. Though recent evidence suggests lopinavir and ritonavir had no clinical effect on SARS-CoV-2, perhaps future application of antiviral drugs should also take into account their effects on the liver [32].

Many infected with SARS-CoV-2 regularly use paracetamol as it is the recommended antipyretic medication. Unintentional overdose with paracetamol contributing to raised ALT cannot be excluded in patients’ non-remitting pyrexia, as paracetamol is a well-recognised cause of fulminant hepatic failure [33]. This also needs to be taken into consideration when evaluating liver injury in these patients.

Several drugs have been trialled on SARS-CoV-2 patients such as hydroxychloroquine and azithromycin with ambiguous results on the virus but possibly exacerbating liver injury [34]. This ambiguity leads to many questions involving the management of SARS-CoV-2 and pre-existing liver disease.

### 3.3. Hypoxic Liver

Sepsis complicating severe COVID-19 illness and hypoxia can also be significant contributing factors [30]. Hypoxic liver injury can be characterised by an increase in transaminases due to an imbalance of oxygen supply [35]. This typically occurs in the elderly with right side congestive heart failure [35]. Though the median age of patients contracting SARS-CoV-2 is 47 years of age, the elderly have proven to be particularly vulnerable, with increasing age an indicator of mortality [7,27]. In the elderly population, it is likely that a rise in liver enzymes, particularly transaminases, is due to pre-existing conditions.

### 3.4. Microthromboses

SARS-CoV-2 has been shown to lead to a hypercoagulable state, therefore increasing thromboembolism risk [36,37,38]. It has recently been reported that in certain patient groups, often younger patients, micro vascular thromboses can cause end stage organ damage and may potentially affect the liver. It is also notable that high levels of alkaline phosphatase have been used as a prognostic value for ischemic stroke patients and in identifying high risk haemorrhagic transformation and are also shown to be high in COVID-19 patients suffered thrombotic events, although in other cases, alkaline phosphatase levels have been normal or very mildly raised [39,40,41].

Results of autopsies from Wuhan province, China, have also shown infiltration of lymphocytes and monocytes in the portal area with microthrombosis and congestion of hepatic sinuses [27]. The liver was described as having hepatocyte degeneration accompanied by lobular focal necrosis and neutrophil infiltration. Though histological features of liver failure and bile duct injuries were not observed in these cases [27].

### 3.5. SARS-CoV-2 in Patients with Pre-Existing Liver Disease

Patients with pre-existing conditions have shown increased susceptibility to SARS-CoV-2. At present, it is unclear to what extent pre-existing liver disease contributes to liver injury seen in SARS-CoV-2 patients. A very recent study conducted in the UK on more than 17 million people has identified pre-existing liver disease as an independent risk factor of death in SARS-CoV-2 infections [42].

For instance, it has been shown that patients with SARS-CoV-2 show an increase of monocyte chemoattractant protein 1 (MCAP1), which is a chemokine known to exacerbate steatohepatitis [34]. A recent short communication describes possible implications for patients with non-alcoholic fatty liver disease (NAFLD) [43]. NAFLD patients, alongside those with metabolic syndrome and type 2 diabetes, are often treated with ACE inhibitors, which have anti-inflammatory and anti-obesity effects. While there has not been a reported effect on mortality of ACE inhibitor drug use, it has been speculated that ACE inhibitors up-regulate the ACE2 receptor and therefore can increase viral load in patients taking these medications [43,44]. NAFLD patients often exhibit increased cytokine levels due to their chronic inflammatory stage. Prins and Olinga suggest that this predisposition, in patients infected with SARS-CoV-2, could expedite the progression of NAFLD to a more aggressive non-alcoholic steatohepatitis [43].

There is suggestion that derangement of liver function should be taken into consideration alongside other physiological values [11,30]. Patients with SARS-CoV-2 have exhibited increased levels of creatine kinase, lactate dehydrogenase, ferritin, C-reactive protein, and myoglobin alongside liver dysfunction, and it has been suggested that liver damage is collateral, caused by induced cytotoxic T cells and the induction of the innate immune response rather than direct injury from the virus itself, as observed with other respiratory viruses [11,16,30,45].

Regardless of the source of injury, it is clear that managing those with pre-existing liver disease needs to be thought out carefully during this pandemic and in future outbreaks of coronavirus infection. These patients are at higher risk of being infected and of more severe COVID-19 disease and should be practising strict social distancing or shielding if they take steroids or immunosuppressive therapies [46]. The British Liver Trust has recently called on the UK government to classify those with extreme liver disease as ‘extremely vulnerable’ [47]. Recent reports suggest that more than 1/3 of cirrhotic patients who developed SARS-CoV-2 died [48]. A new international registry developed between the University of Oxford and the University of North Carolina has shown that those with decompensated cirrhosis are at most risk and are calling on hospitals to routinely test patients with deranged liver function/enzyme results for SARS-CoV-2 so early observation and treatment may prevent further deterioration. The British Liver Trust also suggests that all patients with decompensated cirrhosis practice social shielding, a step up from social distancing, even though it is not yet part of the formal guidance [47].

Boettler et al. have published comprehensive recommendations for management and surveillance of patients with liver disease throughout the SARS-CoV-2 outbreak [28]. This paper now forms the official position of the European Association for the Study of Liver and the European Society of Clinical Microbiology and Infectious Disease [49]. They suggest prioritization of outpatient clinics, inpatient admission depending on presence of certain risk factors, reducing exposure through social distancing (remodelling waiting areas, reduction of waiting times, reduction of face to face contact through telemedicine), and carefully considering the benefits of patient care weighed against the risk of infection.

## 4. Disease Severity in the Immunocompromised and Transplant Patients

Under ordinary conditions, organ transplant recipients and those on immunosuppressants are at high risk of infection due in particular to the suppression of T cell response, making their susceptibility to SARS-CoV-2 and prognosis, if infected, unclear. On one hand, it has been postulated that reduction of systemic inflammation by immunosuppressants could improve outcome for COVID-19 patients as the severity of inflammatory response can be an indicator of prognosis [50]. However, it is also a case that immunosuppressed individuals tend to have a higher viral load, take longer to shed the virus, and may show more severe clinical symptoms with a poorer prognosis [51].

Zhu et al. reported on 10 SARS-CoV-2-positive renal transplant recipients in Wuhan, China [51]. All were admitted to hospital with significant progressive pneumonia. The severity of pneumonia in this group was recorded as greater than their infected family members and others in the local population. In accordance with Influenza A/HINI guidance, calcineurin inhibitors were stopped in seven patients for nine days and in one patient for 12 days [51,52]. Within this group, there was no acute renal graft rejection, and all patients eventually recovered from COVID-19, though it took longer for them to become SARS-CoV-2-negative than their infected family members [51]. They attributed the length of infection but eventual recovery to the hypothesis that long-term immunosuppression might delay viral clearance and prolong the course of disease but avoid fatal pneumonia caused by a hyperimmune response [51].

Another study of 90 SARS-CoV-2-positive transplant patients in New York City also described reducing antimetabolites, steroids, and/or calcineurin inhibitors in 55 patients [53]. Pereira et al. categorized patients as mild (outpatient care only), moderate (admission as general inpatient), or severe (mechanical ventilation, admission to intensive care unit, or death) [53]. Within this group, 24% presented with mild disease, 46% moderate, and 30% severe. As with other studies, advanced age and comorbidities were associated with disease severity [7,27,39,40,41]. Type of transplant and time of viral infection after transplant were not statistically significant factors [53]. Laboratory values were similar between moderate and severe cases, though albumin was lower in the severe group [53].

At present there is little data regarding the use of immunomodulatory agents such as tocilizumab or sarilumab when trying to suppress the ‘cytokine storm’ in these patients [53]. Pereira et al. noted that 14 patients receiving 1–3 doses each of tocilizumab and 16 patients receiving bolus steroids showed no adverse outcomes at the time of their publication [53]. They also noted that while all biomarkers of inflammation were elevated, procalcitonin was the only marker which differed between moderate and severe disease and suggested that the chronically immunosuppressed may undergo a uniquely dysfunctional inflammatory response to SARS-CoV-2. This was further supported by Lippi et al., who showed that high levels of procalcitonin can be a predictor of severe COVID-19 syndrome and potentially related to secondary bacterial infection [54]. From this study there were no confirmed cases of thromboembolic complications or organ rejection [53].

Many epidemiological reports regarding treatment and prognosis of COVID-19 syndrome are based on the general population who would have had healthy immunity before viral infection, thus overlooking important data for immunocompromised patients [53]. Many such patients present with atypical signs and symptoms leading to missed diagnosis, late presentation, and worse prognosis [50]. At the time of this publication, no significant conclusions have been drawn regarding the outcome of COVID-19 in patients in receipt of immunosuppressive therapy. More research into cytokine activation, T cell signalling and migration, and viral clearance are needed [53]. The postulated anti-inflammatory benefits of immunosuppression should be balanced against the possibility of inhibiting anti-viral immunity by delaying viral shedding and possible organ rejection for those patients having undergone transplant [50].

## 5. Vaccination for SARS-CoV-2

Ultimately, a vaccine against SARS-CoV-2 will be key in preventing spread of virus and loosening social restrictions, but many factors need to be considered in the development of a vaccine so as not to increase innate immune response, increase likelihood of autoimmune diseases, or further DILI.

Vaccinations are costly and usually take years to complete stringent animal and human trials before being made available to the public. However, in an epidemic or pandemic situation, the scientific community faces increasing pressure to rapidly respond with an effective vaccine. In previous epidemics such as Ebola, H1N1, SARS, and MERS, vaccine development was never completed due to the epidemic ending and funds being reallocated [55].

In the context of this review, it is important to highlight that one possible side effect of vaccinations could result in liver damage. Vaccines with the greatest potential, in pandemic situations, are RNA- or DNA-based vaccines [55]. These vaccines do not need to be cultured or require fermentation, they avoid risks of working with live pathogens, and can specifically encode key antigens without also coding for other toxins, but they are not without risks [55,56].

There are no approved RNA vaccines to date, as toxicity cannot always be predicted from animal studies due to species differences between human and animals [55]. Some effects seen in previous RNA-based vaccinations have been pancreatitis, lactic acidosis, liver steatosis, nerve damage, and death [55]. Liver toxicity was reported in preclinical studies using RNA therapy for Crigler–Nayjor syndrome, and in an RNA-based rabies vaccination trial, an increased and deleterious inflammatory response ended the trial [55]. This is possibly due to type 1 interferon induction by RNA, which is known to induce autoimmune diseases [55]. DNA-based vaccines have also been implicated in inducing an innate immune response through toll-like receptor (TLR) 9 and non-TLR activation [56].

## 6. Conclusions

SARS-CoV-2 is a novel coronavirus known to cause respiratory infections with severity ranging from mild cold- and flu-like symptoms to fatal pneumonia. While respiratory based, if severe, it can cause dysfunction of other organs such as the kidneys and liver. It is likely that the liver injury seen in SARS-CoV-2-positive patients is multifactorial and the result of a combination of inflammatory response, sepsis, hypoxia, microthrombotic events, DILI, and viral damage. Pre-existent liver disease is an independent risk factor of death in SARS-CoV-2 related infection, and severity of liver damage most likely correlates with COVID-19 disease severity. Nevertheless, abnormalities in the liver function tests of these patients, without pre-existing liver disease, may have prognostic significance and predict adverse outcomes. Patients with chronic liver disease and in particular those on immunosuppressive therapies including liver transplant recipients should be particularly careful and managed according to internationally accepted guidelines regarding strict social distancing or shielding.

## Figures and Tables

**Table 1:** Liver enzyme abnormalities in COVID-19 disease vary and reflect the degree of inflammatory response, direct biliary injury by the virus, the presence or absence of ischemia/microthromboses, and possible drug-induced liver injury. Hypoalbuminaemia and high transaminases levels are associated with poor prognosis.

		Albumin	Transaminases	GGT	Bilirubin	Alkaline Phosphatase
COVID-19	Severe liver injury from inflammatory response (cytokine storm)	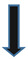	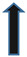	Variable	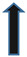	Variable
	Drug induced liver injury	Variable	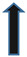	Variable	Variable	Variable
	Direct biliary injury	Variable	Variable	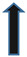	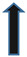	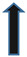
	Ischemia/microthrombosis	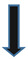	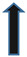	Variable	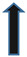	Variable
